# Folic acid deficiency as a modifiable risk factor for anastomotic leak in patients undergoing colorectal cancer surgery

**DOI:** 10.1007/s13304-025-02273-3

**Published:** 2025-06-04

**Authors:** Andrea Chirivella-Fernandez, Javier Rivera-Castellano, Ester Ramírez-Caballero, Samuel Morales-Díaz, Luciano Delgado-Plasencia

**Affiliations:** 1 University of La Laguna, San Cristóbal de La Laguna, Spain; 2 Department of General and Digestive Surgery, Hospital Universitario de Canarias, Islas Canarias, Ofra, s/n, La Cuesta, La Laguna, 38320 Santa Cruz de Tenerife, Spain

**Keywords:** Surgical dehiscence, Folic acid, Sarcopenia, Colorectal cancer, Postoperative morbidity

## Abstract

Sarcopenia predicts negative outcomes in colorectal surgery, including anastomotic leakage (AL). Folic acid (FA) is essential for cellular processes such as DNA synthesis and tissue repair, which may indicate that it could impact anastomotic healing. The objective of this study was to evaluate the association between preoperative blood levels of FA, sarcopenia defined radiologically by psoas density, and postoperative outcomes in patients undergoing resection for colorectal cancer. A prospective study was conducted on patients undergoing oncological colorectal resection with anastomosis between June 2022 and November 2024. FA levels and the average psoas density at the L3 level on computed tomography (CT) were analyzed. Postoperative complications were recorded. Of the 250 patients that were analyzed, 12% had low FA levels (< 2.7 ng/ml) and 16% were diagnosed with sarcopenia. Furthermore, low FA levels were associated with a higher risk of sarcopenia diagnosis [RR 4.63 (95% CI 2.26–9.49)], and radiological sarcopenia was associated with an increased risk of AL [RR 1.98 (95% CI 1.3–2.83)]. Low FA levels are associated with an increased risk of sarcopenia, therefore FA deficiency could be considered a modifiable risk factor for sarcopenia and, consequently, its supplementation preoperatively could reduce the risk of anastomotic dehiscence in colorectal cancer patients.

## Introduction

Sarcopenia is defined as the loss of muscle mass or lean mass associated with ageing and is considered a marker of frailty that correlates with increased morbidity and mortality after surgery [1, 2]. Muscle atrophy can occur under any condition of inactivity, including ageing or chronic pathologic conditions such as myopathy, cachexia, obesity, and malnutrition. Computed tomography (CT) allows for both quantitative and qualitative muscle assessment, thereby enabling the preoperative identification of sarcopenia [3].

Myosteatosis can be defined by the quality assessment of the muscle and has been correlated with low radiological muscle density, measured in Hounsfield Units (HU). According to recent studies the density value that defines myosteatosis varies depending on the patient’s sex [3]. In addition, low muscle density or myosteatosis is an indicator of radiological sarcopenia. This condition has been associated with an increased risk of postoperative complications in gastrointestinal cancer patients and has been studied as a predictive factor for anastomotic leakage (AL) [2, 3].

Clinical diagnosis of sarcopenia is based on the evaluation of muscle strength and functional capacity. This assessment is easily available but tends to be more subjective, depending on the clinical skills of the assessing professional. In contrast, radiological sarcopenia measures muscle mass through imaging, making it more precise but requiring an imaging test, which is usually more expensive but is already performed in colorectal cancer patients and thus does not incur additional costs. Even though clinical evaluation provides useful information on how muscle loss affects the patient’s ability to perform daily activities and their quality of life, radiological sarcopenia can help identify early changes in muscle mass that may not be noticeable through clinical evaluation.

To reduce the risk of AL, we must provide our patients with resources to ensure adequate muscle mass before surgery. Several biomarkers related to muscle mass have been identified, such as carnitine, HDL, LDL, and folic acid (FA) [4–6]. Regarding the latter, an association has been found between clinical sarcopenia and FA deficiency [7], but no studies have yet correlated this with radiological sarcopenia or increased AL.

FA (vitamin B9) is involved in DNA synthesis, making it a key factor for muscle fibre regeneration and growth, especially after an injury. In addition, it contributes to the metabolism of methionine and histidine, increasing ATP production and serving as the primary cellular energy source. FA also helps reduce homocysteine, a byproduct of metabolism that, in excess, generates oxidative stress and muscle damage. Furthermore, FA deficiency has been linked to neuromuscular diseases [8].

The aim of this study is to evaluate the association between FA deficiency, radiological sarcopenia, AL risk and postoperative complications in patients undergoing resection for colorectal cancer treatment.

## Methods

A prospective cohort study was conducted on patients undergoing colorectal cancer surgery with curative intent by colorectal surgeons from June 2023 to November 2024.

**Inclusion criteria:** Patients aged 18 years or older.

**Exclusion criteria:** Minimally invasive transanal surgery (TAMIS), abdominoperineal amputation, colostomies, emergency interventions, delayed emergency interventions, and patients unable to be followed up after discharge.

### Outcomes

#### Primary objective

To assess the risk of sarcopenia in patients with low FA levels, as well as the risk of anastomotic dehiscence in patients with a radiological diagnosis of sarcopenia.

#### Secondary objectives

For the evaluation of the secondary objectives, the sample was divided into sarcopenic and non-sarcopenic patients to compare the rate of complications, reintervention, readmission, surgical site infection, and mortality between both groups. In addition, the risk of experiencing severe complications (CD > 2) was assessed.

### Data collection

The epidemiological variables collected in the study included age, sex, preoperative physical status according to the American Society of Anesthesiologists (ASA), body mass index (BMI), weight loss, preoperative serum haemoglobin (HbPre) and FA levels, and the type of surgical intervention (right hemicolectomy, extended right hemicolectomy, transverse colon resection, left hemicolectomy, splenic flexure resection, sigmoidectomy, anterior resection of the rectum, low anterior resection of the rectum and ultralow resection of the rectum).

The FA values were analysed as a binary variable: normal levels (> 2.7 ng/ml) and insufficient levels (≤ 2.7 ng/ml) based on preoperative blood analysis.

The CT scans of all patients were reviewed using Centricity imaging software, and the density of both psoas muscles at the L3 axial cut was measured to define radiological sarcopenia in patients with mean psoas density below 34.4 HU in men and 34.1 HU in women [3].

### Follow-up

Patients were followed until discharge, evaluating postoperative complications (< 3 months): AL, reintervention, mortality, readmission, and surgical site infection.

### Statistical analysis

Statistical tests were conducted using SPSS 26.0. Descriptive data was presented as mean (± standard deviation), median, or percentage (%), as appropriate. Cross-tabulation was used to assess the association between FA, radiological sarcopenia and AL. Relative risks (RR) are presented with 95% confidence intervals (CI). A p value ≤ 0.05 was considered significant.

## Results

### Epidemiological characteristics

Of the 329 patients who underwent surgery during the study period, 250 patients were included after applying the exclusion criteria. Demographical data for the patients is shown in Table 1.

**Table 1:** Epidemiological characteristics of the patients

	Sarcopenia, *n* = 40	Non-sarcopenia group, *n* = 210	*p* value
Age (years), mean (SD)	72.48 (10.61)	69.42 (10.60)	0.096
Sex (male)—*n* (%)	32 (80.0%)	122 (58.1%)	0.009
BMI, mean (SD)	27.25 (5.27)	26.99 (5.11)	0.771
Weight loss, mean (SD)	2.56 (4.13)	2.05 (4.81)	0.535
HbPre, mean (SD)	12.61 (1.79)	12.94 (1.72)	0.268
FA levels mean (SD)	3.67 (2.17)	7.36 (4.12)	< 0.001
ASA—*n* (%)			
I–II	26 (65.0%)	138 (65.7%)	0.931
III–IV	14 (35.0%)	72 (34.3%)	
Location *n* (%)			
Right hemicolectomy (including right hemicolectomy, extended right hemicolectomy, transverse)	16 (40.0%)	84 (40.0%)	0.932
Descending (including left hemicolectomy, sigmoidectomy, Splenic angle)	11 (27.5%)	63 (30.0%)	
Anterior resection of the rectum (including ultralow anterior resection of the rectum, low anterior resection of the rectum)	13 (32.5%)	63 (30.0%)	

*SD* standard desviation, *BMI* Body mass index, *HbPre* Preoperative Haemoglobin, *FA levels* Folic acid level, *ASA* American Society of Anesthesiology

Right hemicolectomy was the most frequently performed surgical procedure in both groups. The surgical approach was predominantly laparoscopic, performed in 174 cases (69.6%), whilst the Da Vinci robotic approach was used in 55 cases (22%), and in the remaining 21 cases (8.4%) an open approach was chosen. Only 7.6% (*n* = 17) of the patients that underwent a minimally invasive approach required conversion to open surgery.

### Radiological sarcopenia

The preoperative radiological assessment of muscle density is shown in Table 2. Sixteen % (*n* = 40) of the patients were diagnosed with radiological sarcopenia according to the CT scan. The mean psoas density in patients diagnosed with sarcopenia was 31.92 HU (± 5.84), and 50.01 HU (± 7.2) in those without sarcopenia, with the difference between both groups being statistically significant (p = 0.049). In the individual evaluation of the right psoas and the left psoas, statistical significance was also observed between the sarcopenia and non-sarcopenia groups.

**Table 2:** Radiological results

Variable	Sarcopenia, *n* = 40	Non-sarcopenia group, *n* = 210	*p* value
HU (mean) (SD)	31.92 (5.84)	50.01 (7.2)	0.049
HU right Psoas	26.38 (5.956)	66.38 (4.125)	0.047
HU left Psoas	31.56 (4.97)	50.02 (7.581)	0.035
Sarcopenia right	32 (80%)	4 (1.9%)	< 0.001
Sarcopenia left	32 (80%)	7 (3.3%)	< 0.001

*HU* Mean of Hounsfield Unit, *SD* Standard deviation

### Folic acid levels

Twelve % (*n* = 30) of the patients had insufficient preoperative FA levels. Of the 40 patients diagnosed with radiological sarcopenia, 60% (*n* = 24) had insufficient FA levels in their preoperative blood tests, meanwhile only 2,4% (*n* = 5) of the non-sarcopenic patients had deficiency in FA levels.

### Anastomotic leakage (AL) and other postoperative complications

Patients with sarcopenia showed a higher prevalence of postoperative complications. The rate of AL was considerably higher in the sarcopenia group, with 40.0% (*n* = 16) of patients affected, compared to only 0.5% (*n* = 1) in the non-sarcopenia group (*p* < 0.01).

In the group of patients with sarcopenia, more severe complications were observed, with a Clavien–Dindo index (CD) > 2 in 40.0% (*n* = 16) compared to 3.8% (*n* = 8) in the non-sarcopenia group. In addition, patients with sarcopenia had a higher rate of reintervention (35.0% (*n* = 14) vs 1.9% (*n* = 4)) (*p* < 0.01) and more readmissions (15.0% vs 2.4%) (*p* = 0.006). The surgical site infection rate was 50.0% (*n* = 20) in the sarcopenia group, compared to 5.2% (*n* = 11) in the non-sarcopenia group (*p* < 0.01).

Mortality in the sarcopenia group was also higher (7.5% vs 1.4%) (*p* = 0.021). The total mortality rate was 2,4% (*n* = 6). The causes of death were abdominal sepsis due to anastomotic dehiscence (*n* = 3), respiratory issues (*n* = 2), acute pulmonary oedema (*n* = 1) (Table 3).

**Table 3:** Complications of the patients

Complications—*n* (%)	Sarcopenia, *n* = 40	Non-sarcopenia group, *n* = 210	*p* value
CD ≤ 2	24 (60.0%)	201 (96.2%)	< 0.01
CD > 2	16 (40.0%)	8 (3.8%)	
Leakage—*n* (%)	16 (40.0%)	1 (0.5%)	< 0.01
Reintervention—*n* (%)	14 (35.0%)	4 (1.9%)	< 0.01
Readmission—*n* (%)	6 (15.0%)	5 (2.4%)	0.006
SSI—*n* (%)	20 (50.0%)	11 (5.2%)	< 0.01
Mortality—*n* (%)	3 (7.5%)	3 (1.4%)	0.021

*CD* Clavien–Dindo index, *SSI* Surgical Site Infection

### Relative risk

Low FA levels were associated with an increased risk of radiological sarcopenia [RR 4.63 (95% CI 2.26–9.49)]. The diagnosis of radiological sarcopenia was associated with an increased risk of AL [RR 1.98 (95% CI 1.3–2.83)] and CD > 2 [RR 5.45 (95% CI 2.18–13.63)].

## Discussion

Clinical sarcopenia is characterised by the loss of muscle mass, strength, and function in older adults. The cohort of our study was formed by patients predominantly of advanced age (69.9 years) and with a moderate prevalence of overweight (BMI = 27.0). Most of the patients were male and classified as ASA II-III, suggesting that the sample is composed by patients with a higher surgical risk due to advanced age and associated comorbidities.

The mean and median psoas density for the entire cohort were comparable to other studies where the same cutoff point was used for the diagnosis of radiological sarcopenia [2, 3]. According to the results of our study, the diagnosis of radiological sarcopenia was associated with an increased risk of AL [RR 1.98 (95% CI 1.3–2.83)] and CD > 2 [RR 5.45 (95% CI 2.18–13.63)]. These data suggests that even the presence of early stages of radiological sarcopenia is associated with an increase in postoperative complications.

FA, or vitamin B9, has been identified as a key factor in musculoskeletal health, and its deficiency is associated with a higher risk of sarcopenia. It is a water-soluble vitamin that plays a role in intracellular DNA synthesis, repair, and methylation, as well as the biosynthesis of nucleotides and amino acids. Skeletal muscle tissue contains the stem cells necessary for myogenesis. After a physical injury, such as surgery, these cells are activated and enter the process of myogenic differentiation. Furthermore, due to its contribution in the synthesis of nucleotides and amino acids, normal levels of FA may have a positive impact on the patient's immune response, which is crucial for preventing postoperative infections and other adverse events. On the other hand, folate deficiency leads to DNA hypomethylation, attenuates muscle cell synthesis, and induces hyperhomocysteinemia, which triggers oxidative stress, inflammation, and a decrease in muscle function, leading to reduced physical performance, strength, and muscle fibre size [8]. The study results associate low FA levels with an increased risk of radiological sarcopenia [RR 4.63 (95% CI 2.26–9.49)]. Therefore, it is advisable to monitor FA levels in patients undergoing colorectal cancer surgery, as it could be considered a modifiable risk factor for AL.

The possibility of detecting early stages of sarcopenia through radiological imaging in patients with the added risk factor of low FA levels would allow for the implementation of measures to reduce the risk of sarcopenia and, consequently, AL, even preventing the development of further stages such as clinical sarcopenia [7, 8].

Exercise is considered the best treatment for sarcopenia, followed by adequate protein intake. However, there are various nutritional supplements that may help improve muscle mass and reduce the risk of anastomotic dehiscence. Preoperative protein supplementation with added FA, in combination with physical exercise, could be a protective intervention by decreasing the risk of low FA levels, sarcopenia, and therefore reducing the risk of AL in patients with colorectal cancer. However, there are no studies that establish the need for protein and FA supplementation in all patients, the most effective dosage, nor the optimal period of supplementation prior to surgery. After the results of our study, we consider it necessary to provide protein supplementation that contains FA in patients with radiological sarcopenia [8–10].

## Conclusions

The results suggest that patients with low preoperative FA levels are at higher risk of sarcopenia, which in turn is associated with an increased risk of AL and higher rates of postoperative complications in colorectal cancer patients. FA supplementation could be an important protective factor to reduce these risks, improving both muscle quality and the postoperative recovery process by decreasing muscle fatigue, weakness, and enhancing physical performance. Therefore, it is recommended to consider the evaluation and supplementation of FA in the preoperative management of these patients, especially those at risk of vitamin deficiency.

## Data Availability

Datasets is presented in the main manuscript.
